# The influence of theory on the formation of the infirmary during antiquity and the Middle Ages in the West

**Published:** 2014-12-10

**Authors:** Efstathios Drampalos, Vasileios Stogiannos, Panagiotis Psyllakis, Mohammad Sadiq, Ioannis Michos

**Affiliations:** 1 Department of Orthopedics, Wrightington Hospital, UK;; 2 Department of History and Methodology of Science, Univerity of Athens, Greece;; 3 Department of Orthopedics, Bristol Royal Infirmary, Bristol, UK;; 4 Department of Orthopedics, Manchester Royal Infirmary, Manchester, UK;; 5 ΄Δ Orthopedic Department, Asklipieion Voulas Hospital, Athens, Greece.

**Keywords:** infirmary, medical theory, antiquity, Middle Ages, West

## Abstract

The modern infirmary is the evolutional product of the dialectic interface between medical theories at each time and the outcome of their application in clinical practice. The infirmary as we know it today did not exist during antiquity, but the different precursors of the modern hospital emerged as a result of the interaction between medical theory and practice. During antiquity the Hippocratic work decisively contributed to the creation of the Asklipieion, an institution with predetermined structure created to heal diseases. Later in antiquity new types of infirmaries appeared along with the evolution of private practice for physicians. Establishment of the first modern hospitals was an outstanding contribution of Islamic medicine during reign of the Islamic Empire. Although there was little progress in the development of medical theory in medieval West, evolution of the infirmary continued and was mostly influenced by Christian religion and charity. In Constantinople large medieval infirmaries were built, but patient care was frequently offered in monasteries by clergymen. Later on medicine and treatment of diseases were taken over by physicians and taught in universities, and medical theory continued on its course of evolution. It is obvious that the modern infirmary is not only a place for treating diseases, but rather the upshot of a series of advancements in science, the relations between people or even countries, and the way humanity perceives its nature and the future. Our research is focused on the interactive relationship between the evolution of medical theory and the infirmary as an institution during antiquity and the Middle Ages with particular emphasis on the Western World.

## Introduction

The modern infirmary is the evolutional product of a process consisting in the dialectic interface between the medical theories about illness at each time and the results of their application in clinical practice. The hospital is the most advanced type of infirmary comprised of a block of buildings and the surrounding environment in urban structure. Hospitals have a particular architectural morphology and functionality, and are built using specialized methods, specifications and materials. In a hospital there are the main spaces for treatment called the wards, as well as theatres, laboratories, and the areas for ancillary use, that is, galleys and warehouses. From the point of view of function and purpose the modern hospital is a place where medicine is practiced in order to cure patients based on approved methods. The present work aims to show that the formation of the infirmary has mainly been influenced by views about illness and health that shaped the relative theory in this area.

The term “infirmary” refers to a place for medical practice and includes the geographic position, topography and morphology of the ground, design and layout, functionality and purpose, equipment, furnishing, tools, type of construction materials used in the structure, and other elements that can influence therapy, diagnosis or prevention of illness. “Medical practice” refers to a set of procedures that are considered to cure or prevent illness or even contribute to research at the time of implementation. The term “theory” is used for an idea, conviction, or recognized and accepted practice related to the medical practices of each time, even if it is not considered medical theory in the modern sense.

The idea of the interaction between medical theory and the formation of the infirmary is quite recent. Even though there have been several articles, publications and lectures regarding medical theory and the infirmary, there is no relevant research that describes the influence of theory on the formation of this institution. 

## Method

Our search did not produce any articles or books focusing on the interaction between medical theory and the formation of the infirmary. The research was conducted in different stages. Textbooks and lectures from the Department of History and Methodology of Science of the University of Athens (UoA) and other relevant departments of Greek universities were consulted. It should be mentioned that one of the main authors was a graduate of the aforementioned department and had the opportunity to formulate the basic idea and organize the research using the insight of experienced lecturers of medical history. For this purpose, works that contained descriptions of the infirmary as an institution or other valuable information were used, even if they were not directly related to the topic of our research. Among them were the works of Hippocrates, Euripides, Homer and other famous personalities such as philosophers, historicists, physicians and so on. In order to collect relevant information, the keywords “infirmary”, “medical theory”, “ antiquity”, “medieval”, “hospital”, “West” and “Asklipieion” were searched on Google, PubMed and Wikipedia. In the course of the study, no articles could be found on the influence of theory on evolution of the infirmary. Therefore, the research question involved the existence of the infirmary in different forms, for instance the ancient Asklipieion, the Roman infirmary or the most recent form, the medieval infirmary. The present paper evaluated the infirmary as an institution as well as medical theories of various historic eras, and aimed to establish a correlation between the two.

**Antiquity**

In ancient Greek mythology we find the first references to treatments for various diseases and this confirms the presence of a theory since theory comes before observation and application (1). In Greek mythology, Melampus was a legendary soothsayer and healer, originally from Pylos, who ruled at Argos. He was the first to cure diseases with medicine and purifications without the divine help (2). Furthermore, Hercules was cured from the mania that the queen of the gods, Hera, had inflicted upon him by drinking an infusion of a herb called Helleborus (3). 

The oldest known theory about illness and therapy revolves around the divine origin of diseases, the therapeutic power of nature, and treatments sent by the gods, and is chronologically placed around the fifth century BC. God Apollo was the doctor of gods and the sun, and his son Asklipios was the doctor of humans. This theory configures the type of infirmary known as “Asklipieion”, blocks of spacious, buildings in the forest with peri styles and gardens, and at the center the temple of god Asklipios or god Apollo. The most famous infirmary of this type is that of Epidaurus, which was built around 320 BC in ancient Greece. In the “Asklipieion” healing procedures were mystic and priesthood was hereditary. The patients entered the infirmary after sunset, took the purifying bath at the area called “Tholos”, offered sacrifice to god Asklipios and finally entered the area called “Avato”. The latter was the sanctuary where the procedure of “Enkoimisis”, a type of therapeutic sleep, was completed. The goal was not just a therapeutic dream, but a course of purification aiming to bring the patient in contact with the divine. During the night the priest-doctor and his assistants, accompanied by a dog or a snake, visited the patient and cured the disease. It is known that Hippocrates formulated a lot of his medical theories based on concepts from the “Asklipieion” (4).

Medicine developed a more rational, scientific and professional status with Hippocrates (fourth- fifth century BC) who adopted the theory of humorism, which was based on the four humors (black bile, yellow bile, phlegm, and blood) and the four temperaments (wet, dry, cold and hot), and through which he attributed illnesses to natural causes (5). According to the theory of humorism the human body is filled with four basic substances, called humors, which are in balance when a person is healthy. All diseases and disabilities supposedly result from an excess or deficit of one of these four humors. For the first time illness was associated with the patient’s predisposition (i.e. altitude, humidity). Therapy was personalized and considered to be the nature’s work, and the goal was to restore the balance of the humors by applying the principle of treating with the opposite. The doctor and the infirmary simply provided the appropriate environment for the patient.

 Furthermore, there were different types of treatment which included: a) diet for acute and chronic illnesses; b) medication (there were almost 400 different kinds); c) surgery (traumatology, orthopedics, and bandaging); d) gynecology; and e) special treatments (cauterization, purification or evacuation of bowels, phlebotomy and exsanguination) (5). In “Hippocratic Collected Works” or in tests of Galen there is no mention of any form of institution or building where patients would be admitted for treatment (6). Considering the nature of popular therapies and the briefly formulated suggestions and recommendations about the organization of the infirmary in a book by Hippocrates entitled “On the Workshop of a Doctor” (7), it can be deducted that the Hippocratic theory decisively contributed to the formation of the infirmary as a place dedicated specifically to therapy. 

According to the above-mentioned book, in the infirmary there should be a room with adequate light (natural or artificial) and special instruments for surgical procedures, with enough room for the patient, doctor, assistants, equipment and the bed. There should also be a room for bandaging and application of splints, which corresponds to the modern plaster room. 

Hippocrates has another work entitled “On the things pertaining to women” (8) that emphasizes the peculiarity of a woman’s nature, indicating that in the Hippocratic infirmary there was a special room for gynecological examination. The nature of the treatments available at the Hippocratic infirmary (6, 7) indicates presence of the following areas in this type of institution: a) a special place for keeping medicine, since there were almost 400 kinds; b) a special place for purification or evacuation of bowels; and c) hygiene facilities such as toilets, bathrooms, water supply systems and a sewer system. Considering all the aforementioned characteristics it is safe to presume that the Hippocratic infirmary was the first rationally organized infirmary in the history of medicine. 

Research on human body using cadavers was mainly performed by Herophilus and Erasistratus in Alexandria for the first time during the third century BC, and continued with the Roman Kelso (first century AD) and Tertullien (second century AD), and the Greek Galen in Rome during the second century AD (9). The advancements in anatomy during the third century AD in Alexandria were due, among other factors, to the support of the ruler Ptolemy I Soter I (283 BC – 367 BC), who permitted the anatomic dissection of cadavers and funded anatomic research. Anatomy probably contributed to the development of proper instruments and medical facilities Such advancements are not directly reported in ancient texts but their emergence is rationally deducted from the necessity for a specific place to perform dissection and house the means for the procedures and practices (instruments, beds and so on). 

Out of the need for medical care for soldiers during military campaigns emerged military medicine, which is first mentioned in Homer’s Iliad. In Iliad there are descriptions of how the sons of Asklipios, Machaon (surgeon) and Podilarios (dietician-pathologist) heal the soldiers’ wounds and cure their diseases. There are no reports about the military infirmary in Iliad, but it is reasonable to presume that it was in the form of a tent (10). 

Later, in the Roman military organization we find medical professionals skilled in the treatment of wounds (known as medici), and there is also an infirmary in each legion consisting of tent-rooms. The medici were regular soldiers who had received some medical training and looked after the wounded and the sick on the march and at “valetudinaria”. The term refers to temporary military hospitals dedicated to treatment of rather serious injuries and ailments (11). 

Establishment of the valetudinaria was a significant contribution of the Roman military system to the Empire’s healthcare structure. The early versions of Roman hospitals were more like “flying military camps” described in De Munitionibus Castrorum (12) as a minimally equipped structure that could accommodate about two hundred men.

The valetudinaria soon evolved into well-equipped structures made of stone and wood that provided medical care to soldiers, slaves, and gladiators. There is little doubt as to the existence of these hospitals, although they may not have been quite so widespread. This is due to the fact that ruins of many of these hospitals were recognized based on the design of building remains, and not through investigation of surviving records or unearthed medical instruments (13). 

 Physicians offered their services initially in infirmary-sanctuaries, like the Asklipieion or the Anfiareion in Oropos and later in academies, like the ones in the Greek island of Kos or in the city of Knidos in Cyprus. Private practice developed gradually and mainly in ancient Rome, where Greek doctors like Galen, the physician of Marcus Aurelius, treated Roman officials (13). Private practice contributed to the formation of a new type of infirmary, a picture of which was unearthed during excavations in Pompeii. The picture shows rooms intended for the doctor’s private usage, including the dining room, kitchen, archive, library, bedroom and the doorkeeper’s room. All these rooms are located around the Atrium. The examination room is connected both to the street and the Atrium. Here the doctor passes most of his time and receives his patients (14, 15). Near the port of Pompeii is the so-called House of the Surgeon, built around the fourth or third century BC, where interesting surgical instruments were discovered (15, 16). 

**The Middle Ages**

During the middle ages of Western history the first modern hospitals were established in the East as a result of advancements in Islamic medicine. . In this contest physicians and scientists of different origins (including India, Egypt, Greece and the Middle East) and cultures were invited at the famous school of Jundishapur (17). When the Arab Muslims conquested the Persian city of Jundishapur in 638 AD, a major center for the dissemination of ancient manuscripts was born (18) playing an important role in the birth of Islamic Medicine (19). Among the accomplishments of this era were: training physicians in basic sciences; establishment of a curriculum for clinical training in internal medicine and surgery, and licensing of physicians (17, 20). Islamic medicine contributed greatly to the development of better hospitals (20, 21). 

There were separate wards for male/female patients and for different diseases in these hospitals together with teaching of medical students. Drugs were provided free of charge. Also, for the first time records of patients were kept and special clothing was given to inpatients. Health services were free, and all patients were given five gold pieces at the time of discharge to help them support themselves until they could go back to work (17). 

 During the Islamic time many hospitals are funded as independent institutions or as religious trusts frequently in the contest of personal charity (17). Later on, these institutions provided the model for hospitals in medieval Europe. By the 14th century AD there were numerous hospitals in the Islamic Empire. The important ones were placed in Damascus (was built in 706 AD), Baghdad, Jundishapur, Cairo, Tunisia, Jerusalem, Morocco and Granada (17).

Hippocrates, Galen and Dioskorides were among the leading figures in Western medicine during the Middle Ages, and a major part of medical research has been dedicated to commentaries on their works. During this era there was little progress in medical theory in the West, although one significant evolution was the emergence of new types of infirmaries. The most important factors involved were: a) Christian charity; b) appearance of monasteries and cathedral institutions in Western Europe; c) the dominant theory about the spiritual nature of illness; d) epidemic diseases; and e) establishment of medical schools and universities.

Christian charity gives birth to the first infirmaries organized in special buildings, with doctors, nurses, use of animals for carrying patients and also care for the companions of the patients. The foundation of the first Christian infirmary is attributed to Saint Basil the Great. Called the "Basilias", this was a city-like complex which consisted of several buildings for different classes of patients as well as lodging for doctors and nurses (22). Justinian also builds several infirmaries in Constantinopole (11). 

 With the predominance of Christianity in Europe health care was passed on to the Church and members of Christian orders, and monasteries took over medical care. For this reason medieval infirmaries were religious institutions where Christian morality was dominant, and their primary goal was to provide food and shelter to those in need (23). After the Christian conquest of Jerusalem in 1099 and during the First Crusade, members of the religious community who took over St. John’s hospital (also known as hospitallers of St. John of Jerusalem) reorganized the infirmary based on the byzantine prototype. Due to the executive position of Jerusalem, St. John’s hospital was known throughout the European Continent. The Hospitallers built several hospitals in Italy and South France. These hospitals had regulations for their function, for instance the presence of at least 4 doctors was mandatory in order to admit patients. Thus the hospital in Jerusalem served as a model for the modern Western hospital and contributed to the transformation of the infirmary into a foundation specialized in the treatment of diseases (5).

In Christianity medicine is considered charity and every Christian is bound to charity. The idea of charity as a religious duty and the need to help the diseased provided the motive to built guesthouses for pilgrims that later evolved into typical Christian infirmaries (14).

During the Middle Ages mysticism prevailed in Christianity, and although the tests of Galen were often taught in academies and universities, medical theory was dissociated from the naturalistic models of Hippocrates and Galen. Diseases were believed to have spiritual causes and could be cured only through God’s will, a concept similar to evangelical descriptions of Jesus Christ’s miraculous cures. Healing could therefore be achieved only through invocation of God’s help and chrism, prayer and imposition of the hands took the place of medicine. 

A circular of the Tour Synod of 1163 entitled “The Church Detests Blood” deprived clergymen of practicing surgery (23). Exsanguinations, teeth extractions and surgery in general were undertaken by barber-surgeons whose shops were transformed to resemble infirmaries. Exsanguinations were also performed in public baths, which were a popular place of therapy (14). 

The epidemics that appeared in the falling Roman Empire and generally in Western Europe during the Middle Ages found the existing medicine inadequate, and, a big part of the urban population was lost. According to the Galenic theory transmission of disease from human to human is not possible because disease has an internal cause, that is, the imbalance of the four humors. Nevertheless, the authorities of big cities took drastic measures in order to contain the epidemics. Consequently, in 1377 the authorities of Ragusa (known as Dubrovnik in modern day Croatia) decided to isolate those who arrived at the city port from areas afflicted with the plague for 30 days in a nearby island. In 1397 the duration of the isolation was extended to 40 days (quaranta, hence the term quarantine). In Milan whenever a new case of the plague was observed in a house, all the residents were locked in and left to die. Although seemingly barbaric, this measure limited mortality from the plague to 15% in Milan, compared to 25% in the rest of Europe and 60% in Florence alone. The quarantine in its modern sense as an institution for disease control through isolation of patients owes its existence to the epidemics of the Middle Ages and the decisions of political authorities of the time (5).

At the height of the plague there were over 200 institutions for treating the diseased in England and Scotland, and around 2000 in France (14). Specialized institutions were also built for the treatment of Hansen’s disease called the Leper Colonies, which were something between infirmaries and places of isolation (14). The medical school of Salerno was founded in the eleventh century by four professors according to tradition: a Latin, a Greek, an Arab and a Jewish master, symbolizing the interaction of these civilizations. In the twelfth century AD universities were established with four faculties of medicine, law, arts and theology in Bologna (1180), Paris (1200), Oxford (1200) and other cities (24). These universities were in fact unions of professors and their main characteristic, as opposed to monasteries and cathedral schools, was the recognition and awarding of degrees (25). Recognition of university degrees started in 1224 when Frederic II issued an edict stating that physicians who wanted to practice in the Kingdom of Naples had to have a degree from the University of Salerno and the approval of the professors (Hippocratic School) (14). In order to obtain a university degree, students had to attend anatomy lectures mainly on Galenic anatomy and perform anatomical dissections of human and animal cadavers. This was similar to the established practice in the anatomy dissection halls of modern universities.

Although Christianity was dominant in medieval Europe, astrology, magic, alchemy and other superstitions were also practiced. The royal touch as a cure for scrofula (tuberculous lymphadenopathy) (23), itinerant lithotomy (removal of stones from the head of psychiatric patients), spells from priests and other healing ceremonies are examples of such superstitions. 

 Towards the end of the 13^th^ century the first pharmacies were founded in Italy. They were designed after the Al-Qairawan hospital and mosque in Tunisia (26), which had been built under the Aghlabid rule in 830. Al-Qairawan was a simple structure but adequately equipped with halls organized into waiting rooms, a mosque, and a special bath. Pharmacies were initially part of monasteries, but later evolved into private stores that physicians frequented not only to buy medicine, but also to meet colleagues and even to examine patients. The chemist himself was often an astrologist and alchemist at the same time (14). Gradually chemists formed a separate guild while they followed the advice of the local doctor and even performed some medical procedures. In this way the pharmacy became a new type of infirmary.

During the Middle Ages insanity was considered a manifestation of “divine punishment” or the consequence of sin, and psychiatric patients were confined to home under the care of their families. These patients were later detained in abandoned towers, fortresses or monasteries where epileptics, criminals and orphans were also held (for example Fools’ Towers in Germany). Such institutions were mostly intended for detention rather than treatment. In London Bethlem Royal Hospital was founded around 1247 AD and devoted to St. Mary of Bethlehem with the initial prospect of reinforcing relations between England and the Holy Land. Around 1403 AD it started hosting ill, insane and elderly people and by the mid 15^th^ century was transformed into an insane asylum, and that is why the name Bedlam refers to a mental institution (27). 

## Conclusion

The infirmary as we know it today did not exist during antiquity and the Middle Ages, but the various precursors of the modern hospital evolved due to the interaction between medical theory and practice. These institutions were influenced by and at the same time influenced the production of new theories about the origins and treatments of diseases. During antiquity the Hippocratic work and the theory of humorism as well as several other books decisively contributed to the creation of the Asklipieion, an institution with predetermined structure and specialized rooms, instrumentation and personnel created to help the diseased. Mobile military infirmaries were set up by ancient Greek and Roman soldiers and along with this kind of infirmary, military medicine also evolved. Later in antiquity new types of infirmaries appeared alongside the private practice for physicians. 

During the Middle Ages the first modern hospitals were developed as a result of the advancements in Islamic medicine. Although there was little progress in medical theory during medieval times, evolution of the infirmary continued. Some crucial elements influencing the process were Christian religion and charity. Large medieval infirmaries were built in Constantinople, but caring for patients was mostly the responsibility of clergymen in monasteries. Later in medieval West priests stopped practicing surgery and gradually physicians and universities took over medicine and treatment of diseases. Medical theory started evolving again and science played a crucial role in the process. Soon in renascence the infirmary and medical theory started on a rapid course of evolution and acquired their modern characteristics. 

Considering all the above it is evident that the modern infirmary is more than a place for treatment of diseases. It is the result of a long process of scientific development, the relations between people or even counties and the way humanity perceives its nature and the future.
